# RETRACTION: Qidonghuoxue Decoction Ameliorates Pulmonary Edema in Acute Lung Injury Mice through the Upregulation of Epithelial Sodium Channel and Aquaporin‐1

**DOI:** 10.1155/ecam/9828231

**Published:** 2026-02-12

**Authors:** Evidence-Based Complementary and Alternative Medicine

RETRACTION: C. Ma, L. Dong, M. Li, and W. Cai, “Qidonghuoxue Decoction Ameliorates Pulmonary Edema in Acute Lung Injury Mice through the Upregulation of Epithelial Sodium Channel and Aquaporin‐1,” *Evidence-Based Complementary and Alternative Medicine*, 2020, 2492304, https://doi.org/10.1155/2020/2492304.

The above article, published online on 27 September 2020 in Wiley Online Library (https://wileyonlinelibrary.com), has been retracted by John Wiley & Sons Ltd. The retraction has been agreed due to an error identified in Figure 4, in which the Sham/ENaC‐α panel appears to share unexpected, repeated elements with the ALI + HDQ panel of Figure 5b in [1].

The authors were unresponsive to the concerns, and after assessment, the results are deemed unreliable. Therefore, the article is retracted.

The authors did not respond to the retraction.
